# Decline in Processing Speed Tells Only Half the Story: Developmental Delay in Children Living with Sickle Cell Disease

**DOI:** 10.3390/children11030277

**Published:** 2024-02-23

**Authors:** Elise Jade Walker, Fenella Jane Kirkham, Anna Marie Hood

**Affiliations:** 1 Developmental Neurosciences Unit, Biomedical Research Centre, UCL Great Ormond Street Institute of Child Health, London WC1N 1EH, UK; elise.walker.17@alumni.ucl.ac.uk; 2 Clinical and Experimental Sciences, University of Southampton, Southampton SO17 1BJ, UK; 3 Manchester Centre for Health Psychology, Division of Psychology and Mental Health, University of Manchester, Manchester M13 9PL, UK; anna.hood@manchester.ac.uk

**Keywords:** sickle cell disease, processing speed, longitudinal, delay, decline, cognition

## Abstract

Children with sickle cell disease (SCD) may experience cognitive difficulties, including slowed processing speed. Thus, we investigated if processing speed changes over time. From 1992–2001, 103 participants with SCD aged 3–16 years (n ≤ 8.99 = 45; n ≥ 9.00 = 58) completed cognitive assessments. MRI was available for 54 participants. Between 1992–2002, 58 participants consented to one or two further assessments. A repeated measures regression using linear mixed-effects modelling determined longitudinal changes in processing speed index (PSI), examining the interaction between age (continuous variable) and timepoint (i.e., assessment 1 or 3) and controlling for MRI infarct status (i.e., no infarct, silent infarct, or stroke). Those aged ≤8.99 and ≥9.00 at first assessment experienced PSI decline. Declines were most prominent for the processing speed coding subtest, with a significant interaction between timepoint and age, *t*(31) = 2.64, *p* = 0.01. This decline may reflect a developmental delay, likely due to disease progression, with slower improvements in processing speed. Although there have been significant improvements in SCD treatments, mostly in high-income countries, processing speed still remains a target; thus, incorporating clinical monitoring of processing speed may help identify delay and allow for early intervention.

## 1. Introduction

Sickle cell disease (SCD) is an umbrella term for a group of inherited blood disorders that cause abnormally shaped red blood cells with a reduced lifespan [1]. SCD causes symptoms including pain, fatigue, cognitive difficulties, and an increased risk of infection and anaemia [1]. Vascular pathology, blood vessel occlusion and the poor capacity of sickled red blood cells to transport oxygen increase the risk of brain injury for people with SCD [2]. Hypoxia/ischaemia occurs when blood oxygen content becomes low, secondary to low haemoglobin and/or oxygen saturation, and cerebral blood flow cannot compensate adequately to maintain the required metabolic rate [3]. There is evidence for an association with silent cerebral infarction (SCI), which often occurs in the deep white matter of the frontal and parietal lobes [4]; if the infarct is large, there may be clinical symptoms and signs of stroke [5]. 

Neurocognitive complications in patients with SCD include stroke and cognitive difficulties [6], including poor attention [7], executive dysfunction (including working memory challenges), EF [8,9], and slow processing speed [10]. In combination with the broader psycho-social effects of living with chronic illness (e.g., time out of school due to pain or hospitalisation), these cognitive sequelae generate specific challenges for school performance [11,12], coping skills and mental health [13], and overall quality of life [14,15] for young people with SCD. To better support young people with SCD through targeted interventions and individualised support, families, schools, and care providers must know about a child’s specific cognitive difficulties and how these may change over time. 

Recently, Hood et al. (2022) highlighted the possible utility of domain-specific cognitive endpoints for assessing treatment effects for children and young people with SCD [16]. Alongside EF, another meaningful cognitive endpoint mentioned by Hood et al. (2022) was processing speed. Many young people living with SCD experience difficulties in processing speed specifically [17]. Processing speed is the time it takes to complete a cognitive task [18]. It changes as a function of development and consists of the time taken to perceive a stimulus, process the stimulus, allocate attention, recall relevant information/instructions, and execute a response [18,19]. 

Used across multiple iterations of the Weschler intelligence tests [20,21,22,23], the Processing Speed Index (PSI) is a standardised score for an individual’s processing speed—typically calculated using two subtests (i.e., Coding and Symbol Search), with an additional optional subtest (Cancellation). In addition to four other cognitive indices (Verbal Comprehension, Visual Spatial, Fluid Reasoning, and Working Memory), PSI contributes to the calculation of an individual’s Full Scale IQ score. For typically developing children, evidence suggests that processing speed (as measured by subtest raw scores) enters a critical period of rapid development from early childhood up until around 7–9 years of age, with increasing differentiation from other cognitive domains into early adolescence [24,25,26]. From mid-adolescence into adulthood, processing speed appears to stabilise [26,27]. As PSI scores are standardised to reflect these developmental changes, we would expect PSI to stay roughly the same throughout childhood, adolescence, and young adulthood. However, for children and young people with a delay (sometimes due to a medical problem) or interruption in processing speed development, PSI might fall below the population average (mean = 100, SD = 15) or their premorbid baseline. Over time, PSI might either improve (recovery of skills and standardised scores that are higher than their baseline), stay the same (consistent performance or small gains in raw scores over time that are not substantial enough to impact standard scores) or decline (loss of skills which manifests as reductions in both raw and standardised scores) [16]. 

Emerging research indicates that young people with SCD show lower PSI scores compared to demographically matched controls, with either consistency or decline in standardised performance with age examined cross-sectionally [10,28]. However, most research on cognition in SCD has been cross-sectional and focused on assessing intelligence (IQ) [29] and EF [30]. A meta-analysis of the few existing studies reported minimal processing speed difficulties for toddlers with SCD, but as processing speed entered the period of rapid development during childhood and into adolescence, there were greater effects of processing speed deficits on overall cognition [31,32]. In the only published longitudinal study of cognitive development in SCD, Wang et al. (2001) found significantly reduced processing speed scaled scores (Coding subtest) between the initial assessment and 10-year follow-up assessment [6]. However, the Wechsler test in use changed (scores were re-coded), and PSI was not calculated because Symbol Search subtest scores were not included [6]. Wang et al. (2001) identified no significant changes for full-scale IQ, which did not appear to be sensitive to declines in individual domains [6,16]. No other published longitudinal research has examined changes in processing speed over time in young people living with SCD. 

Processing speed difficulties experienced by young people living with SCD may be a mediating factor for other cognitive difficulties, including EF [33], where tasks are timed [10] and processing speed may relate to white matter integrity, as in the general adult population [33]. Border zones in deep white matter and cortical structures are at increased risk for ischaemia and infarction in patients living with SCD [34], including in the deep white matter of the frontal and parietal lobes associated with processing speed [4]. In fact, areas of hyperintensity [17] and abnormal white matter integrity on diffusion tensor imaging [10,35] have been associated with reduced processing speed in young people living with SCD. As such, processing speed may be a valuable endpoint for assessing changes in cognition over time and possible treatment effects.

Given that processing speed deficits are frequently observed, infarct status can influence processing speed, and these difficulties may have an increased impact with age, the goal of the present study was to longitudinally assess whether processing speed significantly declines in children and young people living with SCD. Specifically, we hypothesised that:

(1) Processing Speed Index (PSI) and individual subtests scores (i.e., Coding and Symbol Search) will be higher for children than for young people living with SCD, providing evidence of decline.
(2) Children with stroke or SCI will have lower PSI and subtest scores than children without infarction detected on MRI.
(3) Children living with SCD will have lower PSI and subtest scores than the normative mean, and there will be an interaction between age and timepoint. Specifically, children will have higher scores at timepoint 1 than at timepoint 3, providing evidence of delay and subsequent decline.

## 2. Materials and Methods

We retrospectively analysed cognitive assessment scores and magnetic resonance imaging (MRI) from a published longitudinal dataset [36] known as the East London Sickle Cell Disease Cohort. The Great Ormond Street Hospital Local Research Ethics Committee granted ethical permission. 

### 2.1. The East London Sickle Cell Disease Cohort

Between 1992 and 2001, 105 participants living with SCD were recruited during their regular haemoglobinopathy clinic appointments at the Queen Elizabeth Hospital for Children (now the Royal London Hospital). Parents/guardians provided informed consent for participants to complete the study. For this larger study, participants aged 16.00 years consented. Caregivers provided consent for those ≤15.99 years and those aged 10.00–15.99 years provided assent. All participants took part in cognitive assessment, and 55 participants living with SCD (53%) also completed magnetic resonance imaging (MRI) at timepoint 1. 

Between 1997 and 2002, participants and caregivers were approached again via telephone and/or during clinic appointments and were asked to complete up to two follow-up cognitive assessments using the same procedure (timepoint 2 and timepoint 3). The mean average number of years between cognitive assessment at timepoint 1 and timepoint 2 was 5 years, and between timepoint 1 and timepoint 3 was 8 years. Data regarding any medical interventions received by participants were extracted from the medical notes.

### 2.2. Exclusion Criteria

To assess processing speed change from childhood through adolescence for participants living with SCD in the present study, participants aged 17.00 years at first assessment were excluded from our analyses. Final analyses included 103 participants at timepoint 1 (1992–2001). Fifty-eight participants consented to testing at the initial follow-up, two participants had died, forty-two participants could not be traced, and one participant declined the study. However, at timepoint 2 (1997–2001), only 17 participants completed cognitive testing. At timepoint 3 (2001–2002), 45 participants completed testing. Therefore, only timepoints 1 and 3 were included in our longitudinal analyses.

### 2.3. Cognitive Assessment 

Participants completed their cognitive assessments with one trained assessor before or after their routine clinic appointment. Each session lasted 1–1.5 h and was completed in a private, quiet room. At timepoint 1, participants completed the Wechsler Preschool & Primary Scale of Intelligence—Revised (WPPSI-R) [23] or the Wechsler Intelligence Scale for Children—Third UK Edition (WISC-III) [20,22]. At timepoint 2, participants completed the WISC-III. At timepoint 3, participants completed either the WISC-III or the Wechsler Adult Intelligence Scale—Third Edition (WAIS-III) [21]. We extracted Full Scale Intelligence Quotient (FSIQ) and Processing Speed Index (PSI) scores from these cognitive assessments for each participant for analyses. 

### 2.4. Magnetic Resonance Imaging 

At each timepoint, participants aged ≥6 years were asked to undergo a magnetic resonance imaging (MRI) scan (Siemens 1.5 Tesla SP400, Forchheim, Germany) producing axial T2-weighted images using a short tau inversion recovery sequence (STIR; TR/TI/TE = 4000/145/85 ms; Slice thickness = 5 mm). Two neuroradiologists blinded to the patient’s clinical history examined scans for infarction; discrepancies were resolved after discussion. Each participant was categorised as having no infarct, infarct with no clinical history of neurological symptoms (SCI), or stroke. MRIs were completed across timepoints between 3.08 years and 4.75 years before or after cognitive testing.

### 2.5. Statistical Analyses

Statistical analyses were conducted in IBM^®^ SPSS^®^ Statistics (Version 27.0) [37] and R (Version 4.0.3) [38]. Descriptive statistics provided an overview of the data, including mean FSIQ and PSI over time in alignment with the developmental change in processing speed in typically and atypically developing populations [39]. Preliminary analyses used independent samples *t*-tests and Chi-squared tests. Repeated measures linear mixed-effects models determined longitudinal change in PSI and subtest (Symbol Search and Coding) scores, assessing significant effects of timepoint, age, and an age-by-timepoint interaction. We also ran the same analyses whilst controlling for infarct status as a factor [40,41]. These analyses were conducted with and without infarct status, as only 52% of the sample had available MRIs. Timepoint 2 was excluded from the mixed effects modelling due to the small sample size (n = 17). A Variance Inflation Factor (VIF) was calculated to consider multicollinearity. For our interaction analyses where infarct status was included, we assessed whether processing speed scores significantly differed for younger (median age 7 years) and older (median age 17 years) children depending on infarct status (i.e., stroke, SCI, or no infarct).

## 3. Results 

FSIQ scores were available for 100% of participants at timepoint 1, and PSI scores were available for 71%; PSI was missing for 27 (26%) participants aged under 6 years at timepoint 1 and who completed the WPPSI-R and 3 (2.91%) older participants who had missing data due to incomplete testing. A total of 81 (79%) participants completed MRI during at least one timepoint. MRI was not available for 22 participants (21%); 7 did not undergo MRI due to being under 6 years at timepoint 1 and were then lost to follow-up; 2 participants completed cognitive assessment but declined to complete MRI; and 13 participants did not complete an MRI at timepoint 1 (either did not consent or did not make/attend their appointment) and were lost to follow-up. Of the participants not followed up for cognitive testing (n = 45), there were no significant differences in age, *t*(101) = 0.05. *p* = 0.96, or sex, χ^2^ (1) = 0.02, *p* = 0.88, at timepoint 1 compared to participants who completed the follow-up assessment. 

For participants included in the present study, at timepoint 1, participants were aged 3–16 years (M = 9.01, SD = 3.68; n = 103); at timepoint 2, participants were aged 9–16 years (M = 12.99, SD = 1.99); and at timepoint 3, participants were aged 10–25 years (M = 16.95, SD = 4.04). 

A summary of participant characteristics, separated by age group at timepoint 1, can be found in Table 1. Most participants were homozygous for SCD (i.e., sickle cell anaemia; HbSS genotype). Just over half of the study sample was male (57%). Children younger than 9 years old had significantly higher IQ than older children, *t*(96.99) = 2.22, *p* = 0.03, *d* = 0.44, with a trend for lower PSI, although both groups (≤8.99 and ≥9) fell within the low average range (Table 1). No child was on hydroxyurea and an investigation of the effect of blood transfusion was not undertaken because of confounding by stroke status.

### 3.1. Longitudinal Change in Processing Speed

The general pattern of results was consistent when children with HbSC were removed; therefore, they were included in all analyses to increase power. 

#### 3.1.1. Linear Mixed-Effects Regression without Infarct Status Included

**Figure 1:**
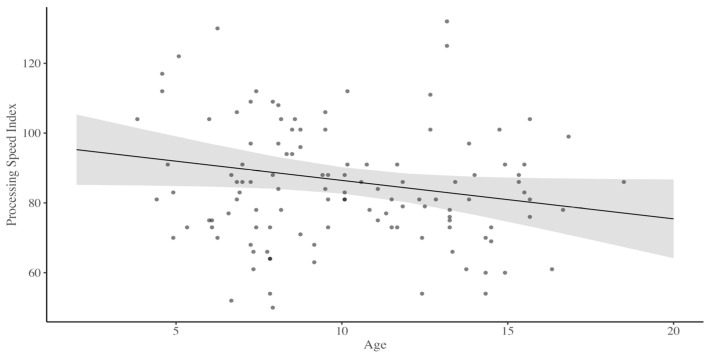
Processing speed index for children living with sickle cell disease by age.

Our first analysis included PSI as the dependent variable. Our model revealed no significant main effect of timepoint, *t*(33) = −1.73, *p* = 0.09, but age approached significance, *t*(81) = −1.94, *p* = 0.06 (see Figure 1). The interaction between timepoint and age was not significant, *t*(33) = 1.03, *p* = 0.31. Our second analysis included the Symbol Search subtest as the dependent variable. Our model revealed no significant main effect of timepoint, *t*(32) = −0.38, *p* = 0.70 or age, *t*(81) = −1.72, *p* = 0.09. The interaction between timepoint and age was not significant, *t*(32) = 0.13, *p* = 0.90. Our third analysis included the Coding subtest as the dependent variable. Our model revealed a significant main effect of timepoint, *t*(37) = −2.76, *p* = 0.01, and age, *t*(84) = −2.59, *p* = 0.01. The interaction between timepoint and age approached significance, *t*(37) = 1.91, *p* = 0.06.

#### 3.1.2. Linear Mixed-Effects Regression with Infarct Status Included

**Figure 2:**
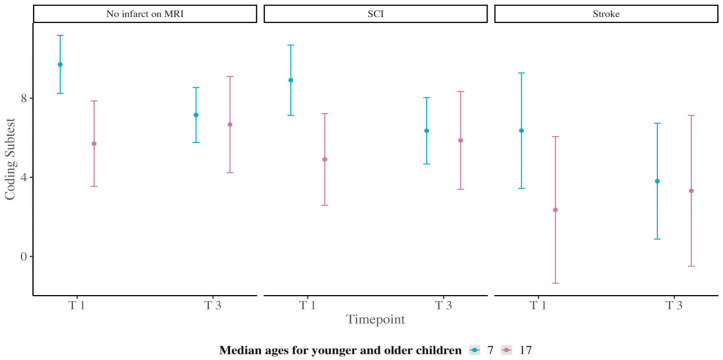
Interaction between timepoint and age, including infarct status for the Processing Speed Coding Subtest.

Our first analysis included PSI as the dependent variable. Our model revealed no significant main effect of infarct status for SCI, *t*(53) = −0.64, *p* = 0.52, or infarct status for stroke, *t*(53) = −1.58, *p* = 0.12, but a significant effect of timepoint, *t*(27) = −2.60, *p* = 0.01, and age, *t*(53) = −2.69, *p* = 0.01. The interaction between timepoint and age was not significant, *t*(27) = 1.72, *p* = 0.10. Our second analysis included the Symbol Search subtest as the dependent variable. Our model revealed no significant main effect of infarct status for SCI, *t*(53) = −0.48, *p* = 0.63, infarct status for stroke, *t*(53) = −0.86, *p* = 0.39, timepoint, *t*(53) = −2.27, *p* = 0.03, or age, *t*(81) = −1.72, *p* = 0.09. The interaction between timepoint and age was not significant, *t*(26) = 0.82, *p* = 0.42. Our third analysis included the Coding subtest as the dependent variable. Our model revealed no significant main effect of infarct status for SCI, *t*(54) = −0.87, *p* = 0.38. However, there was a significant main effect of infarct status for stroke, *t*(54) = −2.12, *p* = 0.04, timepoint, *t*(31) = −3.58, *p* = 0.001, and age *t*(54) = −2.84, *p* = 0.006. The interaction between timepoint and age was also significant, *t*(31) = 2.64, *p* = 0.01. This interaction indicated that younger children living with SCD had higher Coding subtest scores than older children at timepoint 1; however, younger children’s scores declined at timepoint 3. This decline was most prominent for children with no infarct on MRI and those with SCI (Figure 2).

## 4. Discussion

In this research, we investigated developmental change in processing speed in children and young people living with SCD. Our analyses found that young children living with SCD have processing speed scaled scores below typically developing children (M = 100) that continue to decline with age. We found that the decline in PSI between the two timepoints was larger for younger children (aged 5–8.99 years) compared to older children and young people (aged 9–25 years), who experienced a decline but at a slower rate. This finding is most prominent for children with an infarct and those with SCI, as younger and older children who had experienced a stroke generally had lower scores at both timepoints. 

Our data supported our first hypothesis, as the Processing Speed Index scores were lower for older children than younger children (see Figure 1). However, this decline was most pronounced and significantly different for the Coding, but not the Symbol Search subtest. Our second hypothesis was also supported; we found that children with stroke or SCI had lower PSI and subtest scores than children without infarction detected on MRI (see Figure 2). Regarding our third hypothesis, we found evidence of delay and subsequent decline for the processing speed Coding subtest (with no significant decline for overall PSI or Symbol Search subtest) as there was a significant interaction between age and timepoint when infarct status was included in analyses. The Coding subtest requires enhanced graphomotor coordination and learning new patterns (e.g., which shape accompanies which number). In contrast, the graphomotor and learning demands for Symbol Search are much lower. Further, previous research has shown that the Coding subtest is particularly sensitive to any type of organic or functional impairment, even for individuals with less neurological damage [42]. This sensitivity may explain the delay and decline in the Coding subtest observed in our sample of children living with SCD.

Typically-developing children demonstrate a consistent pattern of age differences in processing speed, with improved raw scores over time. This phenomenon has been demonstrated across a wide range of tasks, indicating that it is a global, fundamental mechanism where children and adolescents have limits in the speed with which they can process information [43]. Considering our research and studies of typical development, our results indicate a developmental delay in processing speed for children living with SCD that worsens over time; they develop processing speed skills at a slower rate. If a child has experienced a medical problem such as a stroke, chronic ear infection, or head trauma, they are at higher risk for developmental delays. Given the presence of stroke and SCI in our sample, this is likely a contributor to the developmental delay observed in the present study. Improving our understanding of processing speed early in life may be necessary for charting cognitive decline or plateau over time in this population. 

Critically, slowed processing speed can underlie other cognitive difficulties—most prominently, executive function (particularly where EF tasks are timed). The decline in PSI found in our study may play an important role in our understanding of lower IQ observed in children living with SCD [44]. Preliminary evidence suggests that controlling for processing speed may eliminate differences in IQ between SCD patients and typically developing controls [10,12]—though conflicting evidence exists for this mediating role of processing speed [45]. Future research must explore the role of processing speed in IQ and executive function for children living with SCD. 

Although our study points to a developmental delay in processing speed for children living with SCD at all ages, a previous study from our group found no evidence of slowed processing speed in toddlers. However, research has indicated that the gold standard tests used for toddlers (e.g., Bayley’s scales) may not be accurate long-term predictors of cognitive function in clinical populations [46]. In fact, a meta-analysis has shown that utilising looking-time-based measures of processing speed provides stronger associations with adult cognitive outcomes [47]. Whether toddlers living with SCD have poorer processing speed than typically developing peers is a critical question for future research; however, what is becoming clear from the current study and previous work is that young children have delayed processing speed. Highlighting that delay occurs within the context of disease progression is not trivial. Shifting our language from decline to *delay with decline* could lead to important changes in our support and intervention strategies.

Within our sample, many participants had experienced a stroke or silent cerebral infarction. Powars et al. (1978) demonstrated that clinical stroke during childhood occurs more often in younger participants (≤7 years). Our data indicated that children and young people with clinical stroke demonstrated the slowest processing speed, followed by children with silent cerebral infarction and then those with no infarct detected on MRI. However, in our regression models, infarct status was not always a significant predictor of change in processing speed over time, with stroke being the only significant factor for the Coding subtest. This statistical finding aligns with previous research [48]. Prussien et al. (2019) reported that individuals living with SCD were at risk for cognitive difficulties regardless of infarct status, with difficulties being more pronounced for participants with stroke than those with SCI, followed by those with no infarct—as we found in our study. Infarct status for SCI may still affect processing speed, and due to the limited number of participants in our sample, it is possible that we were underpowered to detect these effects. Another possibility may be an interaction effect between the psychosocial effects of chronic illness (including time out of school) and the possible subclinical pathophysiological processes that occur alongside disease progression. It will be important for future research to consider the potential impact of infarct status for SCI on processing speed development with larger samples whilst incorporating psychosocial factors to understand if processing speed is slowed for those with cerebral infarction compared to all children living with SCD. 

Our finding that following delay, the decline in processing speed scores over time was larger for the younger children provides evidence for the role of increased disease burden over time. Specific mechanisms that might contribute to the observed decline in processing speed include chronic hypoxic exposure alongside episodes of cerebral infarction, which may accumulate with the critical childhood period of rapid processing speed development to produce the observed delay. It is probable that as these clinical risk factors accumulate, they influence neurological function. Prior work indicates that hypoxic exposure reduces neurotransmitter turnover rate, which, in turn, affects the ability to remain vigilant and respond correctly to visual stimuli, i.e., processing speed [49]. 

Subsequently, many potential SCD symptom-related targets warrant consideration for the observed decline. Beyond infarct status, differences over time in cerebral blood flow [50] have been demonstrated compared to typically developing children. Anaemia severity [51], the volume of white matter hyperintensities [17], and any disturbance of white matter integrity [10,35] also predict processing speed in people living with SCD, whilst obstructive sleep apnoea, which is common in children with SCD, has also been associated with slowed processing speed [52]. A reduction in functional connectivity of subcortical networks [53] and progressive brain atrophy [54] have been found in other research. Future longitudinal research could consider these clinical risk factors and their association with processing speed to better understand if they may be related and potentially assess the role of disease-modifying therapies (e.g., hydroxyurea) unavailable during our study period. 

### 4.1. Limitations 

Drawing on retrospective longitudinal data was particularly useful for these analyses due to the lack of available longitudinal cognitive data for SCD cohorts. Nonetheless, there were multiple challenges that using such data generated. 

The recruitment of our sample began over 20 years ago, and current disease-modifying treatments were unavailable. Therefore, we were not able to consider treatment status in our analyses. Further, the mean PSI in our sample was relatively low compared to the current literature on SCD, where PSI values between 90–100 have been observed for participants with SCD [9,10]. This difference likely reflects the improvement in treatment for SCD in the last 20–30 years. Many children and young people are now being treated with hydroxyurea [55,56,57,58] or chronic blood transfusions [9], with some receiving successful curative treatments [59,60] that may improve cognitive outcomes [9,55,56,57,58,60,61]. Hydroxyurea may improve cognition as it increases foetal haemoglobin, which reduces haemoglobin S polymerisation and the “sickling” of red blood cells [62]. Chronic blood transfusions likely improve cognition as they increase the oxygen-carrying capacity of the red blood cells and decrease the proportion of sickle haemoglobin (HbS) relative to haemoglobin A (HbA) [62].

However, we still believe our findings have a place in the modern era, as treatments are not available to most children living with SCD, particularly in areas with the highest disease burden (e.g., sub-Saharan Africa), or outside of large medical centres. Further, although improved, cognitive performance, particularly processing speed and executive abilities, still remain lower than peers and siblings. Thus, one possible contribution of this work might be providing medical providers with additional reasons to encourage the uptake of disease-modifying treatments.

Our findings highlight the need to investigate whether the identified processing speed decline, particularly in the Coding subtest, is present in a current cohort of children receiving more advanced treatment methods. Future research should investigate neuroprotective treatment effects on processing speed development in young people with SCD longitudinally [9,16,58,60,61], with consideration of the role of non-verbal and visuo-spatial abilities in processing speed outcome measurement [55,56,57]. This will allow for clinically relevant replication of our longitudinal research design.

The sample size was also significantly reduced at timepoint 2, so these participants were excluded from regression analyses. Further, by grouping participants by age and infarct status, our analyses were underpowered to detect smaller effects. However, it is possible that because MRI did not always occur immediately before or after cognitive testing, some SCI or stroke may have been missed. Additionally, because of our smaller sample size, we did not include available information in analyses (i.e., genotype). Larger sample sizes may allow researchers to overcome these challenges and help to consider patients in more specific SCD subgroups. 

Finally, a decline in standard scores for any cognitive measure generally reflects slowed developmental progress relative to peers. Assessing raw scores allows researchers and clinicians to determine decline without considering normative data (i.e., identify if a young person is demonstrating a plateau in abilities or is improving but not at an expected rate) [41,63]. Due to different assessment tools (e.g., WPPSI and WISC) being used for children of different ages (within and across timepoints), we could not explore the utility of raw score data and are limited in the conclusions we can draw about why a decline in standardised scores was observed. This highlights the importance of utilising assessment tools which cover multiple age ranges and can be used longitudinally with limited practice effects [28].

### 4.2. Future Directions 

Despite slowed processing speed being reported frequently in previous research, our study is one of only a few that has assessed these challenges longitudinally in children living with SCD. Monitoring the long-term trajectory of processing speed is an important next step for clinical practice to assist in identifying emerging cognitive difficulties, monitor treatment effects, and indicate the risk of hypoxic exposure for early intervention. With technology constantly evolving, such regular monitoring is becoming more feasible. Clinicians may utilise cognitive assessments that can be used across a larger age range, such as the NIH Toolbox (ages 3–85 years), so that there is no need to administer a different version of a test to enable age-appropriate assessment (e.g., WPPSI to WISC). Thus, raw scores can be considered longitudinally. Researchers may use raw scores for longitudinal group comparisons or cross-sectionally during shorter-term clinical trials [63]. In clinical practice, the fact that the NIH toolbox only takes 35 min to administer can reduce the burden for patients, increasing the likelihood that they return for repeat cognitive testing. Our previous work demonstrated that incorporating brief, easily administered, relatively inexpensive screening measures into the clinical care of children with SCD is warranted. The current study supports including short processing speed tests in these screening batteries.

Finally, children living with SCD require self-management skills, e.g., the purposeful performance of specific learned tasks, activities, and behaviours [64,65]. However, cognitive dysfunction, such as impairments in processing speed, can impede an individual’s ability to successfully self-manage their chronic disease [66]. Additionally, processing speed challenges can affect academic achievement [67]. Therefore, our findings indicate that healthcare providers and educators should adapt their support approaches when processing speed deficits are identified.

## 5. Conclusions

Our findings demonstrated that children living with SCD experience developmental delays in processing speed within the context of disease progression that worsens with age. This decline was the largest for children aged ≤8.99 years, with an interaction demonstrated between timepoint and age for the Coding subtest. Critically, these findings point to *delay with decline*. There are many potential reasons for our results, including clinical risk factors that could not be treated due to the limited availability of disease-modifying treatments at the time of data collection. To build upon our findings and contribute to improving clinical care, researchers may consider more longitudinal research and consider processing speed and physiological indicators of neurologic injury cross-sectionally in studies of children and young people living with SCD. Clinicians should continue to monitor the cognitive outcomes of children living with SCD using appropriate longitudinal measures to better understand processing speed to capture developmental delay early and, thus, have the capacity to intervene and prevent subsequent decline. 

## Figures and Tables

**Table 1:** Participant characteristics at timepoint 1 are separated by age (over and under 9 years).

	≤8.99 Years (n = 58)	>9 Years (n = 45)	Total (n = 103)	*p* Value
Age				
M (SD)	6.2 (1.5)	12.6 (2.2)	9.01 (3.7)	
Baseline Intelligence Quotient				0.03
M (SD)	85.67 (12.54)	80.11 (13.01)	83.18 (12.99)	
Range	49–111	55–107	49–111	
Baseline Processing speed Index				
M (SD)	88.55 (17.09)	81.57 (15.78)	85.47 (16.77)	0.08
Range	50-130	54-132	50-132	
Sex				0.73
Female	25 (43.10%)	17 (37.77%)	42 (40.78%)	
Male	33 (56.90%)	28 (62.22%)	61 (59.22%)	
Genotype				0.68
HbSC	9 (15.52%)	5 (11.11%)	14 (13.59%)	
HbSS	45 (77.59%)	38 (84.44%)	83 (80.58%)	
HbS Thalassaemia	4 (6.90%)	2 (4.44%)	6 (5.83%)	
MRI Infarct Status				0.40
No Infarct	20 (34.50%)	20 (44.44%)	40 (38.83%)	
SCI	11 (18.97%)	10 (22.22%)	21 (20.39%)	
Stroke	13 (22.41%)	6 (13.33%)	19 (18.45%)	
No MRI available	14 (24.14%)	9 (20.00%)	23 (22.33%)	

M = mean; MRI = magnetic resonance imaging; n = number of participants; SCI = silent cerebral infarct; SD = standard deviation; *p* value = significance for difference between those aged ≤8.99 Years and those aged >9 Years.

## Data Availability

The data presented in this study are available on request from the corresponding author. The data are not publicly available due to concerns over confidentiality.
